# Prediction of Masked Chafer, *Cyclocephala pasadenae*, Capture in Light Traps Using a Degree-Day Model

**DOI:** 10.1673/031.006.3601

**Published:** 2006-10-27

**Authors:** Carlos A. Blanco, Gerardo Hernández

**Affiliations:** ^1^ U. S. Department of Agriculture, Agricultural Research Service, Southern Insect Management Research Unit. 141 Experiment Station Road, Stoneville, Mississippi 38776; ^2^ CINVESTAV, Sección de Teoría y Metodología de la Ciencia. Apartado Postal 14-740, 07000 Mexico D.F., Mexico

**Keywords:** turfgrass, degree-days model

## Abstract

In order to obtain information on the biology of the masked chafer *Cyclocephala pasadenae* (Coleoptera: Scarabaeidae), and to determine the date when 50% of the population is captured in light traps, field data were obtained during 4 years in Albuquerque, New Mexico. Capture of the 50% of the masked chafer population occurred approximately during the third week of July, of this one-generation per year insect. To reduce the need for intensive sampling and to obtain a predictable model for the capture of this pest, data were analyzed using trapezoidal numerical integration to estimate both a lower threshold and degree-days to predict the 50% capture date. A mathematical model based on field data accounted for the influence of natural environmental conditions on development, and predicted 50% capture dates within 1–4 days of what was actually observed from the field. The difference between predictions from field data is smaller than using estimates from laboratory-controlled experiments. The model presented here could serve as an accurate estimator of the appropriate timing to implement control measures of this important turfgrass pest.

## Introduction

Turfgrass is the most important vegetative component of many urban landscapes. In Albuquerque, New Mexico, it covered 30.7% of the city's area in 1989 (Blanco-Montero et al. 1995). Large amounts of water, fertilizers and pesticides are used to maintain this area in satisfactory conditions. The biotic factors that exert the greatest maintenance pressure in this semi-arid geography of New Mexico are pests, particularly the masked chafer *Cyclocephala pasadenae* (Casey) (Coleoptera: Scarabaeidae), which often is controlled with insecticides. Although several non-pesticide alternatives have been developed against this pest (Blanco-Montero 1993; Blanco-Montero and Hernandez 1995), scouting to determine the abundance of arthropods in space and time is one of the most important and time-consuming factors in their control. Populations of subterranean insects such as masked chafers are particularly difficult to assess, therefore integrated pest management programs could be greatly improved with an accurate and reliable predictable method.

The development rate of ectothermic organisms is affected by biotic factors such as nutrition, disease, and growth as well as by abiotic conditions such as humidity and an optimum temperature range (Sharpe and DeMichele 1977; Taylor 1981; Wagner et al. 1984; Higley et al. 1986). Accumulation of temperature over time has been successfully used as a predictor of insect development, and researchers and pest managers have used this tool to predict the occurrence of a number of pests (Pruess 1983; Wagner et al. 1984; Higley et al. 1986; Chang 1988; Harari et al. 1998; Bostanian et al. 1999). Traditionally, degree-day methods start with experiments that establish the relation between temperature and the number of days necessary for a species to complete its development given a fixed set of temperature values. Many publications have focused on numerical techniques to estimate degree-days (e.g. linear, max-min, sine, bias-corrected sine, etc.). Some researchers have suggested that all these techniques produce essentially similar results, whereas others have made important observations on the limitations of degree-day theory (Arnold 1959; Sharpe and DeMichele 1977; Taylor 1981; Wagner 1984; Higley et al. 1986; Hagsturm 1991, Hagsturn and Millken 1991; Manel and Debouzie 1997; Bryant et al. 1999). Among these limitations, the crucial one is that constant temperatures used in laboratory experimental procedures disregard natural variations in temperature occurring under field conditions.

Degree-day techniques have been used in integrated control methods (Wegner and Niemczyk 1981), and the use of these techniques is of particular importance when insecticides with shorter residual activity are used as part of the control strategy. When dealing with subterranean pests in turfgrass, it is crucial to time the application of pesticides to coincide with the most vulnerable insect developmental stage, and in this sense a degree-day predictor model could be an extremely useful tool. *C*. *pasadenae* copulate after adult flight and eggs are deposited in the thatch which hatch a few days later. Larvae begin to feed on the soil-thatch profile, an area where most of the insecticides accumulate. Having this soil-thatch profile protected will ensure that this insect would come into contact with the insecticide during its most susceptible stage - the early larval instar. Therefore, predicting peak adult activity time would make control efforts more efficient in terms of labor and time, and it might help reduce the number of applications necessary for the control of *C*. *pasadenae.*

The time of peak flights for this pest usually occurs when 50% are captured in light traps. To estimate this peak using the degree-day method, light trap capture data were used that take into account both normal temperature variations and environmental conditions that influence development. The resulting trap-capture predictor model, based on field-collected data, could predict insect activity and reduce the amount of labor involved in monitoring *C*. *pasadenae.*

## Materials and Methods

### Field collections

Light traps (20W-fluorescent white bulb, installed at 1.8-m height) were used to collect *C*. *pasadenae* in 3–5 golf courses and well maintained parks throughout the Albuquerque, New Mexico, metropolitan area between May and September, from 1990 to 1993. The number of collections varied between years. These capture sites were established at a distance of 1.2 to 12.7 km from the Albuquerque International Airport, where air temperatures used in this study, were recorded by the National Weather Service (National Oceanic and Atmospheric Administration information [NOAA]) every 3 hours. Traps were checked 3 times a week during low capture days (before July 01), and daily when capture increased (July and August).

### Mathematical techniques

To establish temperature thresholds in laboratory-controlled experiments, temperatures are usually maintained constant, and then a relation is established between temperature and the number of days necessary for a species to complete its development (*d_e_*). In most cases, experimental data can be approximated by a straight line, when data are plotted to relate temperature, *T*, and 1/*d_e_*. Clearly, the variable 1/*d_e_* never vanishes, since that case would require an infinite value for *d_e_.* However, the prolongation of the line that approximates such points does intersect the abscissa axis. The value for the temperature that corresponds to this interception is used as the lowest threshold of temperature, denoted by *T_L_*. The equation of this line is generally written as

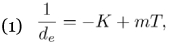

where *K* and *m* are positive constants estimated by linear regression analysis. Since *T_L_* is the value of temperature for which 1/*d_e_* = 0, we find that *T_L_* = *K*/*m*. The equation that allows computation (and that actually defines) the degree-days is now used:

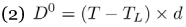

where *D^0^* denotes degree-days after *d* days at constant temperature *T.* The computed value for *T_L_* is substituted to get

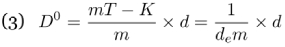

The last equality results after using (1). From here we concluded that the degree-days for development completion of a given species, *D_e_^0^*, is given by *D_e_^0^* = 1/m (we simply set *d* = *d_e_* in (3)). With this result, to predict above ground emergence date the number of degree-days must be added, day by day, over the estimated threshold to reach the value *D_e_^0^*.

As stated earlier, experimental methods are traditionally used to estimate two parameters: the lower threshold of temperature (*T_L_*) and the degree-days for emergence (*D_e_^0^*). A computer program was developed to estimate these parameters using field data, assuming that temperature is the main factor in the development process, and using (2) to compute degree-days, even when field temperatures were not constant. Two consecutive years (1990 and 1991) were considered for which the date for 50% of the population captured by all traps could be estimated.

Since it was assumed that the number of degree-days needed to complete the development of a given species (starting from a given standard date, say, 01 January) for both years must be the same, the computer model began with an arbitrary low estimate of the lowest threshold of temperature *T_h_*, say 10.5° C. The model computed the degree-days for each year by converting the original temperature data *t_i_*, to a new value *T_i_*, by defining *T_i_*= *t_i_* - *T_h_*, whenever *t_i_* was greater than *T_h_*, and zero otherwise. Then the trapezoidal numerical approximation was used to calculate the degree-days from 01 January to 10 July for 1990, and from 01 January to 19 July for 1991.

The program replaced *T_h_* by *T_h+1_*, and so forth, and for each hypothesized value it computed the difference between the number of degree-days obtained for both years. Through this method the lowest temperature threshold was defined as the one that produced the minimal difference, and the degree-days were set as the average of the computed values for both years. Obviously, the program can be modified to increase the hypothetical value of the lower threshold by any fraction of temperature degrees, giving any desired precision.

Finally, the predicted dates of 50% of population capture in light traps were compared for all these experiments and variations with actual sampled emergencies in 1992 and 1993.

## Results and Discussion

**Figure 1.**
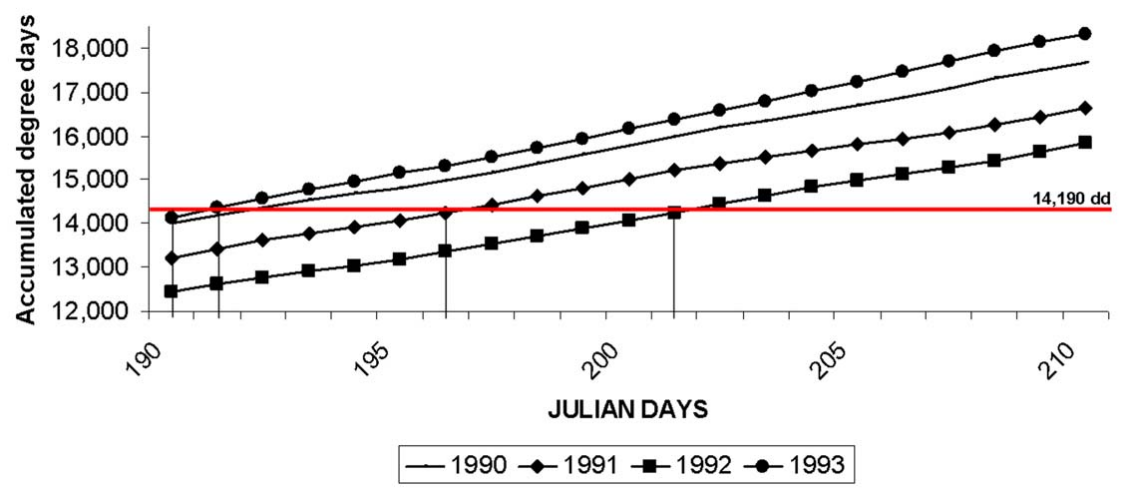
Accumulated degree-days for a lower threshold of 10.2 °C. Horizontal line corresponds to the amount of degree days (14,190) that minimizes differences between the years 1990–1991 for 50% capture of *Cyclocephala pasadenae* in light traps.

**Figure 2.**
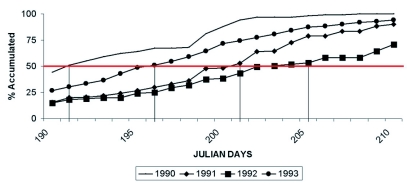
Accumulated percent capture of *Cyclocephala pasadenae* in light traps in Albuquerque, New Mexico

After several manipulations with temperature values to reduce the discrepancy between the calculated and observed dates as much as possible, the minimum threshold for *C*. *pasadenae* was modified to 11.3° C and the predicted 50% capture was 191 days for 1990 and 197 for 1991. This new threshold was then applied to 1992 and 1993 air temperature data (see Figures 1 and 2) obtaining 201 days for 1992 (1 day off) and 191 days for 1993 (4–5 days off). To prove the consistency and value of this methodology, 1992 and 1993 data were used and the same set of results were obtained, resulting in an optimal minimum threshold of 10.2 °C. This compares favorably with the theoretically-calculated minimum threshold of 10.8° C obtained with the southern masked chafer, *Cyclocephala immaculata* Olivier, that is very similar in terms of size and volume to *C*. *pasadenae* (Potter 1981).

The values obtained by this method provide an accurate date for pest control decisions for practical purposes of forecasting the capture of 50% of *C*. *pasadenae.* Studies done with similar techniques (Goodenough et al. 1983; Mailloux et al. 1988; Manel and Debouzie 1995; Bostanian et al. 1999; Esker and Nutter 2002) have found similar results, with discrepancies up to 15 days. In Figure 2 the accumulated capture follows a logistic curve. Therefore, 50% of the captured population corresponds to the maximum rate of increment in capture; that is, higher capture values are obtained around this date, mostly immediately after it.

Finding a slightly different lower threshold and a lower number of accumulated degree days from what was reported for *C*. *immaculata* (Potter 1981) could have resulted from the following changes. First, air temperature values were used instead of soil temperature. This decision was taken for practical reasons, since air temperature data are easier to obtain through weather services agencies. The second factor is that development from egg to adult emergence is not considered as recordings started January 1, when the masked chafer has already reached the larval stage. Finally, emergence from pupae to adults is not recorded, as the population of flying adults is simply recorded. The basic principles of the degree-day method were used, but with the aim of predicting peak capture dates. Precisely because the experiment began in January and eggs were laid the previous year at different dates, larvae were at different developmental stages. This might be a factor that would account for deviations from our predicted values. The uniqueness in using the method described here is that it does not involve the need to maintain organisms under constant temperatures or artificial diet. Its advantage is that it can utilize field data obtained during a minimum of 2 years or 2 non-overlapping insect generations, overcoming the potential difficulties of rearing insects under laboratory conditions.

Environmental factors other than temperature, especially humidity, may play an important role as well (Potter 1981; Higley et al. 1986; Mondino et al. 1997; Rodriguez del Bosque 1998). Although Potter (1981) found that rain occurrence was strongly associated with *C*. *immaculata* light trap captures, more so than temperature, when field-capture data for *C*. *pasadenae* were plotted with rain information from the NOAA a clear pattern was not obtained (data not included). Perhaps the fact that the beetles in this study were collected in areas under daily irrigation (≈ 1.2 cm/night), a negligible amount of rain, as occurred during this study (4.24 and 6.0 cm as monthly average for July and August respectively for 1990 to 1993), was not enough to produce a physiological and/or behavioral response. Similar observations have been also made by Stone (1986) and Rodríguez-del-Bosque (1998) studying other scarab beetles.

Degree-day theory has proven its effectiveness in predicting the development and emergence of numerous species at constant and variable temperatures (Sharpe et al. 1976). Its application to forecast adult captures of subterranean pests is another useful application of this tool. However, other undetermined factors that are as yet unclear, interfered with getting a more precise estimation of the adult capture when this information was generated in the field and extrapolated into a model.
